# Efficacy of PD-1 & PD-L1 inhibitors in older adults: a meta-analysis

**DOI:** 10.1186/s40425-018-0336-8

**Published:** 2018-04-04

**Authors:** Rawad Elias, Anita Giobbie-Hurder, Nadine Jackson McCleary, Patrick Ott, F. Stephen Hodi, Osama Rahma

**Affiliations:** 1 Sections of Hematology Oncology and geriatrics, Boston University School of Medicine, Boston, MA USA; 2 Department of Biostatistics & Computational Biology, Dana-Farber Cancer Institute, Boston, MA USA; 3 Department of Medical Oncology, Dana-Farber Cancer Institute, Harvard Medical School, Boston, MA USA

## Abstract

**Background:**

Immune checkpoint inhibitors targeting PD-1/PD-L1 pathway demonstrated promising activities in variety of malignancies, however little is known regarding their efficacy in adults aged ≥65 years.

**Methods:**

We conducted a systematic review and a study-level meta-analysis to explore efficacy of ICIs based on age, younger vs older than 65 years. We included in this analysis randomized controlled phase II or III studies in patients with metastatic solid tumors that compared efficacy of PD-1 or PD-L1 inhibitors to a non-PD-1/PD-L1 inhibitor. Aggregated estimates of overall survival (OS) and progression-free survival (PFS) are based on random/mixed effects (RE) models to allow for heterogeneity between the studies.

**Results:**

Initial search identified 53 articles, 17 were randomized controlled trials that compared nivolumab, pembrolizumab or atezolizumab to chemotherapy or targeted therapy. Only 9 trials reported hazard ratiios (HR) for OS based on age and were included in this meta-analysis. Out of those studies seven reported HR for PFS but only 4 studies included subgroup-analysis based on age for PFS. The overall estimated random-effects HR for death was 0.64 with 95% CI of 0.54–0.76 in patients ≥65 years vs. 0.68 with 95% CI of 0.61–0.75 in patients < 65 years. The overall estimated random-effects for HR for progression was 0.74 with 95% CI of 0.60–0.92 in patients ≥65 years vs. 0.73 with 95% CI of 0.61–0.88 in patients < 65 years.

**Conclusions:**

PD-1 (nivolumab and pembrolizumab) and PD-L1 (atezolizumab) inhibitors had comparable efficacy in adults younger vs ≥ 65 years.

## Background

The advent of Immune Checkpoint Inhibitors (ICIs) changed the landscape of cancer treatment. Efficacy of PD-1 and PD-L1 antibodies has been established in a wide spectrum of solid and hematological malignancies. [1–10] However, although cancer is predominantly a disease of older adults, the clinical efficacy of ICIs in this population has not been specifically assessed. [11, 12] Published literature suggests that aging-associated immune changes may have an impact on the activity of checkpoint inhibitors, including PD-1 and PD-L1 inhibitors. [13] Cytotoxic CD8+ T cells in older adults were found to have decreased TCR (T cell receptor) diversity, reduced proliferative capacity, and increased sensitivity to apoptotic signals compared to younger adults [14–16] In some studies, aging was associated with decreased expression of CD28 on the surface of CD8+ T cells which leads to decreased immune activation. [17–19] Expression of CD57, a marker of senescence, was found to be increased on the surface of cytotoxic T cells of older adults contributing to a diminished anti-tumoral immunogenic response. [20, 21] In addition, the levels of perforin and granzyme, both essential for T cell’s cytotoxic activity, were lower in older adults compared to younger individuals. [22] Interestingly, expression of PD-1 was found to be increased on T cells of older adults and its blockade did not restore T cell activity to the same extent as in younger adults [22–24] Our understanding of the efficacy of PD-1 and PD-L1 antibodies in older adults is limited due to underrepresentation of this patient population in prospective clinical trials due to concerns about the safety profile of the investigated agents. [25] Consequently, we conducted a systematic review and a study-level meta-analysis to explore efficacy of ICIs based on age, younger vs older than 65 years.

## Methods

### Search strategy and selection criteria

**Fig. 1:**
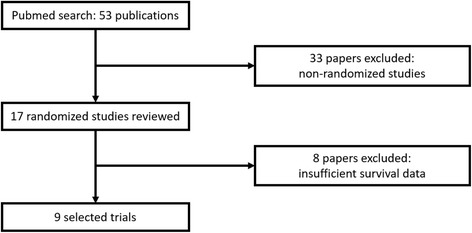
Flow diagram of study inclusion

We performed a Pubmed database search from January 2009 to December 2016 using the medical subject headings (MeSH) terms “pembrolizumab”, “nivolumab”, and “atezolizumab”, the only Food and Drug Administration (FDA) PD-1/PD-L1 ICIs approved at the time this review was conducted. Search was done using the filter “clinical trial”. The language was restricted to English. We then performed additional searches of Web of Science, ASCO meeting database, and ESMO meeting database using the same terms. We reviewed the “Drugs @FDA” database for randomized studies that did not report number of patients aged ≥65 years enrolled on the trial or subgroup analysis for overall survival (OS) by age (younger vs ≥ 65 years). Studies meeting all of the following criteria were included: (1) Randomized controlled phase II or III studies in patients with metastatic solid cancer (2) Studies comparing efficacy of PD-1 or PD-L1 inhibitors to a non-PD-1/PD-L1 inhibitor (3) Subgroup analysis for survival using a hazard ratio (HR) based on age performed in study or available in FDA label review. The selection process is shown in Fig. 1. Studies involving use of ICIs in hematologic malignancies were excluded from this meta-analysis.

### Data extraction

Data extracted from eligible studies included: (1) Study characteristics (first author, year of publication, study name, design, phase, arms, National Clinical Trial (NCT) number (2) Study population (total number of randomized patients, total number in each arm, total number of patients younger than 65 years, total number of patients ≥65 years, number of patients younger than 65 years in each arm, number of patients ≥65 in each arm, median age, age range, mean age (3) HR for OS and for PFS (4) HR for OS and for PFS based on age subgroups (younger vs ≥ 65 years). In the case of trials that did not include survival subgroup analysis by age, we reviewed each published trial’s supplement and the FDA medical and statistical review available on the “Drugs @FDA” database.

### Statistical methods

Aggregated estimates of OS and progression-free survival (PFS) are based on random/mixed effects (RE) models to allow for possible heterogeneity between the studies. Forest plots were used to summarize and visualize the HR with 95% confidence intervals (CI) for each study and for the aggregated estimates from the RE models. For studies reporting separate HR estimates for 65–75 and > 75 years, a combined estimate (≥ 65 years) was created using random effects modeling, and the combined estimate was used in the meta-analysis for that study. Chi-squared *p*-values from Cochran’s Q statistic assessed study heterogeneity. Analyses were conducted using the “Metafor” package in R software (Version 3.2.3, The R Foundation for Statistical Computing). Statistical significance is defined as *P* < 0.05; there were no corrections for multiple comparisons.

## Results

### Search results and patient characteristics

We identified 53 studies that matched our basic selection criteria of clinical trial involving one of the FDA-approved PD-1 and PD-L1 agents. Thirty-six non-randomized studies were excluded. Seventeen studies were reviewed, 8 out of those 17 studies were excluded as they did not contain OS subgroup analysis by age. Final analysis included 9 studies: (6 phase 3 studies, 2 phase 2 studies, and one phase 2/3 study). Among the 9 studies included in the analysis, 5 investigated nivolumab, 2 investigated pembrolizumab, and 2 investigated atezolizumab. Tumor types included metastatic non-small cell lung cancer (NSCLC) in 5 trials, melanoma in 2, renal cell cancer in 1, and head and neck cancer in one trial. Characteristics of each trial are presented in Table 1. All studies reported a subgroup analysis based on age except for the POPLAR study [26] for which HR were obtained from the FDA medical review of atezolizumab (Biologic License Application (BLA) 761,041).

**Table 1:** Characteristics of included studies. Abbreviations: NSCLC (non-small lung cancer); S-NSCLC (squamous non-small lung cancer); NS-NSCLC (non-squamous non-small lung cancer); RCC (renal cell cancer); H&N (head & neck); NR (not reported); Q (every); W (weeks)

	Study Name	Drug	Phase	Malignancy	First line	Arm 1	Arm 2	Arm 3	Patient’ number	Age median	Age range	Age mean	n (%) < 65 y	n (%) ≥ 65 y
Rittmeyer 2016 [33]	OAK	Atezolizumab	3	NSCLC	N	Atezolizumab 1200 mg Q 3 W	Docetaxel 75 mg/m^2^ Q 3 W		850	64	33–85	63	453 (53)	397 (47)
Fehrenbacher 2016 [26, 34]	POPLAR	Atezolizumab	2	NSCLC	N	Atezolizumab 1200 mg Q 3 W	Docetaxel 75 mg/m^2^ Q 3 W		287	62	36–84	61.5	174 (61)	113 (39)
Brahmer 2015 [5]	Checkmate-017	Nivolumab	3	S-NSCLC	N	Nivolumab 3 mg/kg Q 2 W	Docetaxel 75 mg/m^2^ Q 3 W		272	63	39–85	63	152 (56)	120 (44)
Borghaei 2015 [6]	Checkmate-057	Nivolumab	3	NS-NSCLC	N	Nivolumab 3 mg/kg Q 2 W	Docetaxel 75 mg/m^2^ Q 3 W		582	62	21–85	NR	339 (58)	243 (42)
Motzer 2015 [4]	Checkmate-025	Nivolumab	3	RCC	N	Nivolumab 3 mg/kg Q 2 W	Everolimus 10 mg daily		821	62	18–88	61.3	497 (61)	324 (39)
Robert 01–2015 [29]	Checkmate-066	Nivolumab	3	Melanoma	Y	Nivolumab 3 mg/kg Q 2 W	Dacarbazine 1000 mg/m^2^ Q 3 W		418	65	18–87	62.7	200 (48)	218 (52)
Ferris 2016 [2]	Checkmate-141	Nivolumab	3	H&N	N	Nivolumab 3 mg/kg Q 2 W	Chemotherapy		361	60	28–83	59.1	248 (69)	113 (31)
Herbst 2016 [8]	Keynote-010	Pembrolizumab	2/3	NSCLC	N	Pembrolizmab 2 mg/kg Q 3 W	Pembrolizumab 10 mg/kg Q 3 W	Docetaxel 75 mg/m^2^ Q 3 W	1033	NR	NR	62	604 (58)	429 (42)
Robert 06–2015 [9]	Keynote-006	Pembrolizumab	3	Melanoma	N	Pembrolizumab 10 mg/kg Q 2 W	Pembrolizumab 10 mg/kg Q 3 W	Ipilimumab 3 mg/kg Q 3 W	834	NR	NR	60.3	467 (56)	367 (44)

### Overall survival

#### Overall comparison

**Fig. 2:**
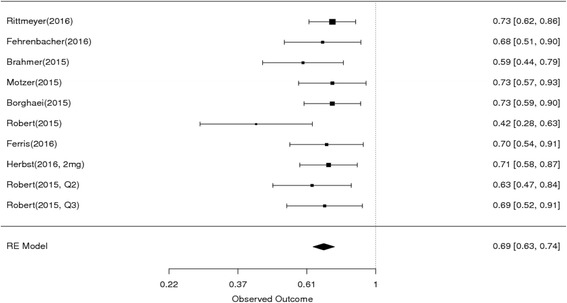
Forest plot for OS. Studies are listed on the left and HR with 95% CI are on the right. Box sizes are inversely proportional to the standard error of the study; therefore, larger boxes indicate greater weight of the trial in the meta-analysis estimation. The HR from the Robert 01–2015^31^ trial is lower than the others, but the weight of the trial is small and does not have great influence

The endpoint of interest is overall survival in studies comparing PD1/PDL1 therapy with chemotherapy/targeted agents. The HR of the individual studies and the combined results based on the random-effects models are summarized in Fig. 2. The ratios presented compare anti-PD1/PDL1 agents against chemo/targeted therapy in the total population. The overall estimated, random-effects HR is 0.69 with 95% CI of 0.63 to 0.74 (*P* < 0.0001). Based on the selected trials, there is evidence of a statistically significant, 31% reduction in the hazard of death with PD1/PDL1 therapy compared with chemo/targeted agents. The chi-squared test for heterogeneity of studies was not significant (*P* = 0.52) suggesting that the reported results of the individual trials are not substantially different from one another.

#### Survival according to age

**Fig. 3:**
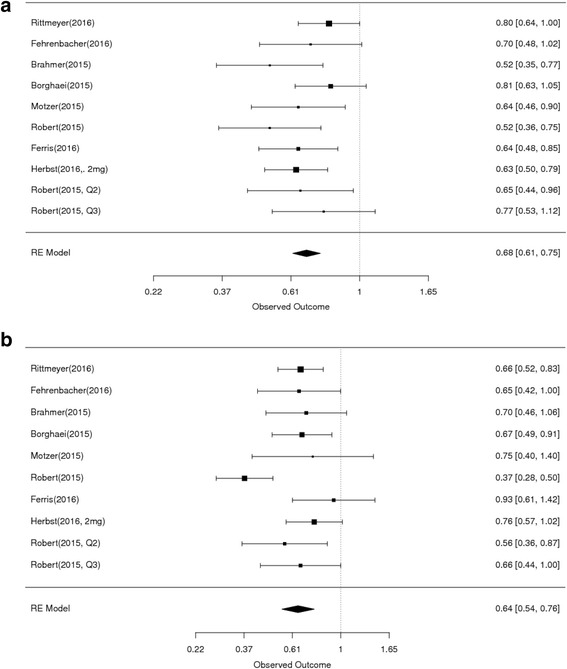
Forest plot for OS for patients less than 65 years (A) and ≥ 65 years (B). Studies are listed on the left and HR with 95% CI are on the right. Box sizes are inversely proportional to the standard error of the study; therefore, larger boxes indicate greater weight of the trial in the meta-analysis estimation

The studies selected for this meta-analysis included a total of 5458 patients. Age range of participants was 18–89 years, and 2324 (42.57%) patients were older than 65 years. The random-effects estimate of the HR of PD1/PDL1 agents compared to control therapy in patients under age 65 is 0.68 (95% CI 0.61 to 0.75) (Fig. 3a). For this subset, there was no evidence of differences between the individual studies (chi-squared *P* = 0.45) in the analysis. The random-effects estimate of the HR for age 65 or older is 0.64 (95% CI 0.54 to 0.76) (Fig. 3b). For this subset, there was evidence of differences in the HR between the individual studies (chi-squared *P* = 0.03), suggesting considerable variability in the reported results among the studies. The comparable hazard ratios for patients 65 and older compared with those under 65 (0.64 vs. 0.68) as well as the substantial overlap of the confidence intervals of the two estimates would indicate that the effects of therapy on survival did not vary for older or younger adults (Table 2).

**Table 2:** Summary of HR for OS by Age

Age	HR (95% CI)
Age < 65 years	0.68 (0.61 to 0.75)
Age ≥ 65 years	0.64 (0.54 to 0.76)

### Progression-free survival

#### Overall progression-free survival

**Fig. 4:**
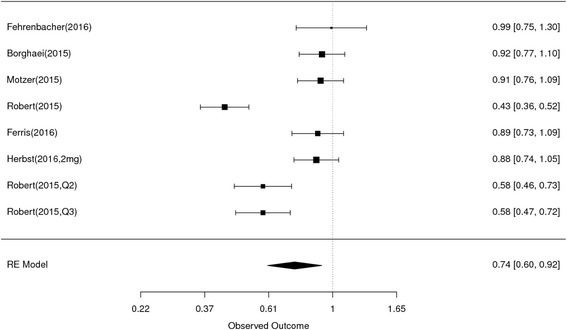
Forest plot for PFS. Studies are listed on the left and HR with 95% CI are on the right. Box sizes are inversely proportional to the standard error of the study; therefore, larger boxes indicate greater weight of the trial in the meta-analysis estimation

The endpoint of interest is progression-free survival in studies comparing PD1/PDL1 therapy with chemotherapy/targeted agents. Among studies included in this analysis, seven had HR for overall PFS including four that reported HR for PFS according to age group. The HR for PFS of the individual studies and the combined results based on the random-effects models are summarized in Fig. 4. The overall estimated, random-effects HR is 0.74 with 95% CI of 0.60 to 0.92 (*P* = 0.006). Based on the selected trials, there is evidence of a statistically significant, 26% reduction in the hazard of a PFS event with PD1/PDL1 compared with chemo/targeted agents. The chi-squared test for heterogeneity of studies was highly significant (*P* < 0.0001) suggesting that the reported HRs of the individual trials are substantially different.

#### Progression-free survival according to age

**Fig. 5:**
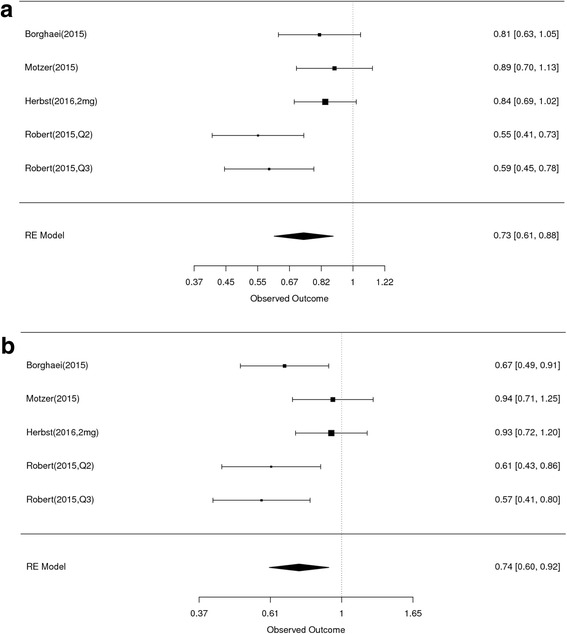
Forest plot for PFS for patients less than 65 years (A) and ≥ 65 years (B). Studies are listed on the left and HR with 95% CI are on the right. Box sizes are inversely proportional to the standard error of the study; therefore, larger boxes indicate greater weight of the trial in the meta-analysis estimation

The random-effects estimate of the HR of PD1/PDL1 compared with chemo/targeted therapy in patients under age 65 is 0.73 (95% CI 0.61 to 0.88) (Fig. 5a), and for patients age 65 or over the HR estimate is 0.74 (95% CI 0.60 to 0.92) (Fig. 5b) For each subset of younger and older patients, there was evidence of heterogeneity between the studies (chi-squared *P* = 0.03 and *p* = 0.05, respectively), suggesting t there is considerable variability in the reported results among the studies within each subset. The equivalent HR estimates for the two age cohorts along with the substantial overlap of the confidence intervals of the two estimates, would indicate that the effects of therapy upon PFS did not vary for older or younger adults (Table 3).

**Table 3:** Summary of HR for PFS by Age

Age	HR (95% CI)
Age < 65 years	0.73 (0.61 to 0.88)
Age ≥ 65 years	0.74 (0.60 to 0.92)

## Discussion

Increased age is associated with changes in the host immunity that could impact effectiveness of ICIs therefore we aimed through this meta-analysis to evaluate the efficacy of PD-1 and PD-L1 inhibitors in adults ≥65 years with advanced solid tumors compared to those < 65 years. This meta-analysis suggests that the impact of PD-1/PD-L1 inhibitors is comparable between adults younger vs. older than 65 years for OS [HR 0.68 (CI 0.61–0.75) vs. 0.64 (CI 0.54–0.76)] and PFS [HR 0.73 (CI 0.61–0.88) vs. 0.74 (CI 0.60–0.92)]. The data were not sufficient to draw any conclusions specific to patients ≥75 years. The number of patients older than 65 years enrolled in PD-1/PD-L1 studies is increased compared to what is usually seen in oncology trials but older adults remain under-represented in cancer clinical trials. [25, 27, 28] This is particularly true for individuals older than 75 years who constitute more than 25% of newly diagnosed cases of cancer every year. [11] Four of the trials included in this review contained HR OS for patients ≥75 years. [4–6, 29] However, out of 2093 individuals included in the analyzed trials only 10% (213) were ≥ 75 year. Accordingly, data was not sufficient to draw any conclusions specific to adults ≥75 years.

Few papers attempted to review this topic but only one was performed at a meta-analysis level. [30] The analysis performed by Nishijima et al..... was based on 9 studies, however among the trials included 4 were with an anti-CTLA4 agents, therefore mixing two classes of ICIs with different mode of action and efficacy profile. Authors showed a comparable OS benefit for ICIs in younger (HR 0.75, 95% CI 0.68 to 0.82) and older adults (HR 0.73, 95% CI 0.62–0.78). It is important to note the age cutoff was non-uniform across selected studies (65–70 years). In addition, authors did not show a statistically significant benefit in terms of PFS for ICIs in adults ≥65 years (HR 0.77, 95% CI 0.58–1.01) vs. a significantly favorable HR in patients < 65 years (HR 0.58, 95% CI 0.40–0.84). Betof et al showed no significant difference in survival benefit with PD1/PDL1 inhibitors according to age in a retrospective analysis of patients treated in two academic medical centers. [31] However, this analysis included only patients with metastatic melanoma and 92.5% were treated with an anti-PD1 agent.

Although it was done at a study-level, our paper constitutes the best level of evidence showing comparable efficacy for checkpoint inhibitors targeting checkpoint inhibitors in adults > 65 years compared to younger patients. Studies included in this meta-analysis, consistent with the majority of clinical trials in oncology, used a numerical age cutoff. An arbitrary age cutoff is not sufficient to characterize “older” adults as aging is a highly variable physiological process. Older individuals are not a homogenous population, therefore measuring variables like functional status and comorbidity is essential to determine the physiologic “age” of an older adult [32]. In addition, older individuals enrolled in clinical trials tend to be adults aged 70–75 years, with good performance status, and a low number of comorbid medical conditions which does not represent the real-life population of older adults with cancer who often have functional limitations and multiple illnesses. Another limitation to our review is that data were obtained partially from FDA BLA review of a particular drug and not directly from the study itself as in the case of POPLAR study. [26]

In conclusion, our meta-analysis showed that improvement in survival associated with the use of PD-1/PD-L1 inhibitors is consistent across age cut-off of 65 years. More data are needed to understand efficacy among those aged ≥75 years as well as tolerance and toxicity of ICIs in older adults overall. Further study is needed including comprehensive assessment of outcomes of significant to older adults, such as functional status and preservation throughout therapy. Geriatric assessment and biomarkers of aging and immune senesce will help to fully understand the impact of ICIs in this growing subset of adults diagnosed with cancer.
